# HOSPICE UTILIZATION AMONG LONG-STAY NURSING HOME RESIDENTS DURING THE COVID-19 PANDEMIC

**DOI:** 10.1093/geroni/igac059.1560

**Published:** 2022-12-20

**Authors:** Xiao (Joyce) Wang, Emma Belanger, Kali Thomas, Debra Dobbs, David Dosa

**Affiliations:** Brown University, Providence, Rhode Island, United States; Brown University, Providence, Rhode Island, United States; Brown University, Providence, Rhode Island, United States; University of South Florida, Tampa, Florida, United States; Brown University, Providence, Rhode Island, United States

## Abstract

**Background:**

The COVID-19 pandemic has had significant impacts on nursing home residents. This study aims to examine how hospice utilization changed among long-stay nursing home residents between January and September in 2020, as compared with the same period in 2019, nationally. Design: A retrospective cohort study of residents present in US nursing homes as long-stay as of January 1st, 2019 and 2020, respectively. A subgroup of residents who died from January to September in each year was also examined. We utilized the Minimum Data and multiple administrative claims data. We compared hospice utilization rate between 2019 and 2020 nationally and by state.Outcomes: This study examined: 1) any hospice utilization among long-stay residents from January to September in 2019 and 2020 respectively, and 2) hospice utilization in the last 30 days of life among the decedent subgroup, which we also tracked as a factor of percent change in mortality rate at the state level.

**Results:**

The hospice utilization rate among long-stay residents was 19.4% in 2019 and 19.7% in 2020. The rate was 27.5% in 2019 and 24.2% in 2020 among the decedent subgroup (χ2=553.1, p< 0.001), although the absolute number of decedents using hospice in the last 30 days of life was higher in 2020 than 2019. Substantial state variation in hospice utilization was observed, mostly following patterns in community-level infections.

**Conclusions:**

Hospice managed to continue service delivery despite many challenges. The pandemic highlights the importance of integrating hospice and palliative care into emergency preparedness planning.

